# Recent Progress on Nanomaterials for NO_2_ Surface Acoustic Wave Sensors

**DOI:** 10.3390/nano12122120

**Published:** 2022-06-20

**Authors:** Livia Alexandra Dinu, Valentin Buiculescu, Angela Mihaela Baracu

**Affiliations:** National Institute for Research and Development in Microtechnologies (IMT Bucharest), 077190 Voluntari, Romania; livia.dinu@imt.ro (L.A.D.); valentin.buiculescu@imt.ro (V.B.)

**Keywords:** nanomaterials for NO_2_ detection, gas sensors, SAW devices

## Abstract

NO_2_ gas surface acoustic wave (SAW)sensors are under continuous development due to their high sensitivity, reliability, low cost and room temperature operation. Their integration ability with different receptor nanomaterials assures a boost in the performance of the sensors. Among the most exploited nano-materials for sensitive detection of NO_2_ gas molecules are carbon-based nanomaterials, metal oxide semiconductors, quantum dots, and conducting polymers. All these nanomaterials aim to create pores for NO_2_ gas adsorption or to enlarge the specific surface area with ultra-small nanoparticles that increase the active sites where NO_2_ gas molecules can diffuse. This review provides a general overview of NO_2_ gas SAW sensors, with a focus on the different sensors’ configurations and their fabrication technology, on the nanomaterials used as sensitive NO_2_ layers and on the test methods for gas detection. The synthesis methods of sensing nanomaterials, their functionalization techniques, the mechanism of interaction between NO_2_ molecules and the sensing nanomaterials are presented and discussed.

## 1. Introduction

Air pollution is the most serious environmental issue, with a significant impact on both human health and the environment. The most harmful air pollutants are particulate matter (PM), nitrogen dioxide (NO_2_), ozone (O_3_) and benzo[a]pyrene (BaP). High concentrations of PM and BaP generally result from the burning of solid fuels used in industry or/and for domestic heating. The O_3_ formation is mainly driven by sunlight. NO_2_ is released by combustion processes [1]. There are two main sources of NO_2_ formation, one caused by human activities, through emissions from cars, industrial processes and power plants, and a natural combustion process produced by forest fires and lightning. NO_2_ is accountable for acidic rains, and ozone formation and is a major environmental cause of morbidity and mortality worldwide, even in low concentrations, if there is a repetitive or long-term exposure [2]. The annual concentration limit of nitrogen dioxide recommended by the World Health Organization (WHO) is 40 µg/m^3^ [3]. Since air pollution is one of the biggest concerns nowadays, new ways to detect and monitor NO_2_ became critical. Intelligent monitoring and wireless sensing are key technologies to the future, paving the way to a better health and quality of life.

In recent years, different types of NO_2_ sensors have been developed based on sensing materials, fabrication technologies, input signals and conversion mechanism or sensor features (such as cost, stability or accuracy). Most studied NO_2_ sensors are metal oxide semiconductor sensors [4,5,6,7], catalytic-type sensors [8], electrochemical sensors [9,10] and acoustic-based sensors [11,12,13].

The operating principle of metal oxide semiconductor sensors is quite simple, mainly based on the change in conductivity of the sensing layer, as the effect of surface doping by the absorbed gas [6]. These kinds of sensors present many advantages including miniaturization, integration with electronics, and low cost. In terms of limitations, metal oxide semiconductor gas sensors include high power consumption, low sensitivity, limited measurement accuracy, and high operating temperature [14].

Catalytic-type sensors detect the temperature rise caused by the oxidation of the gas, which is converted using a standard Wheatstone Bridge-type circuit into an output voltage signal proportional to the gas concentration [8]. The catalytic sensors are simple to operate, have low cost, and have proven technology with high reliability and predictability. The limiting factors in catalytic sensors technology are air or oxygen requirements for sensor operation and the poisoning/inactivation of the catalyst due to contamination (by lead, chlorine or silicon).

The electrochemical sensors allow the gas to diffuse to an electrode through a porous membrane, where it is either oxidized or reduced. The main advantages of the electrochemical sensors include low power consumption, miniaturization and low cost. However, they operate in a limited temperature range and have moderate selectivity [9,15].

SAW sensors use the interconversion of electrical and mechanical energies via the piezoelectric effect. The operating principle of these sensors relies on changes in the propagation characteristics of the mechanical wave due to the interaction between the waves and gas molecules. The process implies three main stages: (i) excitation of the acoustic wave in the piezoelectric substrate, (ii) modulation of both amplitude and phase characteristics as an effect of the sensing process, and (iii) re-conversion of the acoustic wave into an electrical signal for successful detection [16,17,18,19]. The acoustic waves’ penetration depth is of the order of the wavelength, so that most of the energy density is confined to the near-surface region, resulting in high sensitivity to surface perturbation. Important advantages of these sensors include fast response, high sensitivity, reliability, low cost and room temperature operation. In addition, the manufacturing process of SAW sensors is compatible with microelectronics technology, offering mass production, high throughput, scalability and the possibility of integration with other devices [20,21,22,23], performing more complex functions. Furthermore, the SAW sensors can operate in a wide range of frequencies (MHz–GHz), which enables intelligent monitoring (wireless operation).

The integration ability of these devices with different receptor materials made them highly suitable in the NO_2_ sensing field. In recent years, nanomaterials such as carbon-based nanomaterials, thin films of metal oxide semiconductors or conducting polymers as gas-adsorption layers were integrated with the SAW devices fabricated for NO_2_ gas sensing due to their ability to increase the performance of the sensor. The selected nanomaterial improves the adsorption capabilities of NO_2_ resulting in enhanced sensitivity of the SAW device. For real-world applications, the development of NO_2_ sensors must take into account several important aspects such as: environmental stability, portability and miniaturization, costs, reduced power consumption, and customized design. The main application targeted for NO_2_ sensors is to identify the pollution level in industrial areas such as power plants and automotive factories.

This paper presents the progress of NO_2_ SAW sensors, providing a deep insight into different types of nanomaterials used as sensitive layers for NO_2_ sensing. The following chapters focus on the design and fabrication of SAW devices, synthesis methods of the nanomaterials used for sensitive NO_2_ detection, techniques utilized for the functionalization process, the mechanism of interaction between NO_2_ molecules and the sensing nanomaterial, and also the performance parameters of several developed SAW sensors, that include the operating frequency, sensitivity, limit of detection, etc. After conclusions, challenges and future prospects are also discussed.

## 2. State-of-the-Art Nanomaterials for NO_2_ Detection

The detection part of a SAW device is the area where a sensing material is immobilized, and changes occur when the gas is introduced into the analysis chamber [24]. The sensing area is the most important element of creating a gas-sensor using a SAW device. For this reason, the choice of an ultra-sensitive material has attracted much attention in the last years.

When functionalizing a SAW structure with a sensing material, the coating technique should also be compatible with the fabricated SAW structures. Several wet chemical methods (Table 1) such as sol-gel, drop-casting, material transfer, and dip and spin coating techniques have been employed for the functionalization of the SAW devices [25]. From all the above, drop-casting is one of the most used techniques for the functionalization of the SAW sensing area, due to the fact that it is easily performed, does not require any equipment and aids the surface modification of individual devices, when optimization protocol is required. Drop-casting is a simplistic coating method that uses few pipette droplets. Nevertheless, for functionalization at the wafer level, this method presents some drawbacks, such as time-consuming activity and ununiform modified area, also because it is performed by the human hand, it cannot be a high accuracy technique. For wafer level functionalization, the spin-coating method is recommended showing time efficiency, uniformity, and method reproducibility. The transfer method is usually used for carbon-based material, especially graphene mono and bilayer, that can be grown using chemical vapor deposition on a metallic substrate [26], and then the growth substrate is removed in an etchant solution followed by fishing the graphene using a different substrate. This method is beneficial for the functionalization of an individual device, such as the drop-casting method, and not for batch level functionalization.

**Figure 1:**
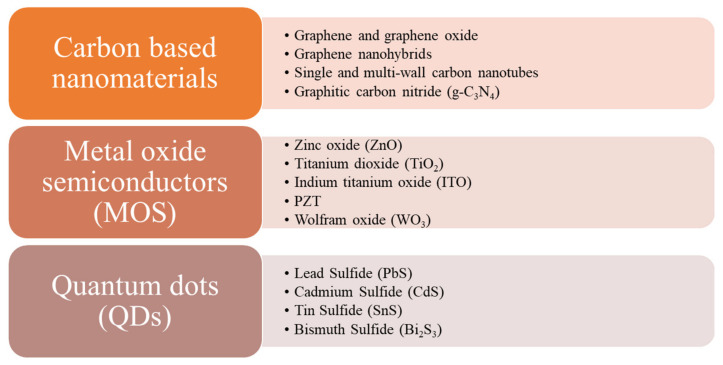
State-of-the-art nanomaterials for NO_2_ detection.

When choosing a gas sensitive material several aspects should be taken into consideration: (i) the presence of free electrons on top of the material, so the gas molecules will be able to interact with the material; (ii) the porous morphology of the material, so the gas molecules will fit into the pores; (iii) a high specific surface area, obtained by introducing different shapes of nanosized material, giving a large number of active sites that will help the diffusion of gas molecules. Several materials have been already exploited, over the years, for sensitive detection of NO_2_ gas molecules, such as: (a) carbon-based nanomaterials, (b) metal oxide semiconductors (MOS), (c) quantum dots, and (d) conducting polymers (Figure 1). For gas detection, the reaction mechanism is also important and it has been intensively discussed for each type of nanomaterial used for NO_2_ detection, in the following subchapters.

### 2.1. Carbon-Based Nanomaterials for NO_2_ Sensing

Since 1985, when Kroto et al., discovered that carbon can exist in different form, such as spheres and buckyballs [27] which was not graphite, anymore, carbon-based nanomaterials have been the most intensively studied nanomaterials in the last decades. C60-C70 carbon spheres/balls were obtained when graphite was evaporated under inert gas. This discovery opened new opportunities for researchers to investigate how carbon can rearrange. Few years later, in 1991, Iijima et al. [28] discovered carbon nanotubes (CNT), when they observed some hollow graphitic tubes by TEM investigations. More than 10 years after, in 2004, Xu et al. [29] have accidentally discovered the carbon dots, during the purification process of CNTs. After the discovery of “graphene” in 2004 [30], carbon-based materials became the backbone of almost every field of science and engineering. From the 2D nanomaterials, graphene (GR) and its derivatives are the most studied carbon-based nanomaterials. GR benefits from several physicochemical properties, but the most important for gas sensing are the following: high stability, large specific area, ease of synthesis and functionalization, low-cost precursors and synthesis routes, and the ability to form porous nanohybrids [31].

Graphene consists of hexagonal networks of the sp^2^ carbon layer, where each two adjacent carbon atoms are strong covalently bonded. Graphene is much stable and of remarkably high quality than graphite and have attracted a significant attention from an experimental and theoretical view [32].

Graphene’s derivates, graphene oxide (GO) and reduced graphene oxide (rGO) are also excellent materials used in sensors’ design, due to enhanced active sites. The modification of structural functional groups makes possible to improve the selectivity aspect of the sensing systems.

GR prepared as ink formulation was used in its reduced form for NO_2_ sensing [33]. GR flakes were exfoliated from graphite under sonication in N-Methyl-2-pyrrolidone (NMP). After the centrifugation of the obtained dispersion, the flakes larger than 1 µm were filtered, to eliminate the clogging issue during jet-printing of the GR ink on top of the sensing area of the SAW sensor. The deposition was temperature sensitive. In 10 min, at 80 °C, 500 drops with controlled dimensions were distributed on the specific area from the SAW device.

For NO_2_ detection, more and more carbonaceous nanomaterials are combined with polymeric materials due to their ability to form a porous surface layer, alloying NO_2_ gas molecules to adsorb rapidly and increase the sensor sensitivity. Polymers represent the family of synthetic and natural macromolecules, which consist of many repeating subunits. The monomeric subunits are bonded to each other by covalent bonds, becoming a very stable compound. Related to the kind of the backbone chain, polymers can be separated into (i) organic polymers, which consist of a carbon backbone, and (ii) inorganic polymers, which comprise other types of elements. In recent years, organic polymers were intensively used due to their electrical properties, and they are known as conducting polymers (CP). They present several advantages such as good mechanical properties, low cost and power consumption, ease of synthesis and miniaturization [34]. Their greatest benefit is the fact that they can operate at room temperature, becoming a much better alternative to MOS materials, contributing also to the ease of operating of the SAW device. For this reason, they have been integrated easily into microfabricated devices for various applications [35]. The two most used syntheses of CP are the electrochemical oxidation and chemical methods, both using as a precursor the specific monomer, and the polymeric structure owns conjugated π-e system, which results in the appearance of conductivity in the material [36]. As a gas detection mechanism, CP encounters an alteration in their electrical conductivity when they are exposed to NO_2_. The most widely used CP in the development of NO_2_ sensors are polyaniline (PANI) [11], poly(3,4-ethylenedioxythiophene) (PEDOT) [31], and polypyrrole (PPy) [37].

Between the CP, PEDOT benefits from increased time-stability and conductivity, making it the most appreciated for marketable applications that require an environmentally stable material [38]. Nonetheless, this CP gets saturated upon exposure to high levels of gas [39], and for this reason, the integration of CP with nanomaterials to form nanohybrids are most recommended in the literature [34], to enhance the sensing properties of stand-alone polymers or carbon-based nanomaterials for NO_2_ detection.

In 2021, a commercially available graphene oxide (GO) was doped with a mixed conducting polymer, PEDOT-PSS to form a hybrid nanocomposite [31] that increased the sensitivity and selectivity toward the sensing of NO_2_. This was due to a large number of functional groups present in the polymer mixture. The nanomaterial was prepared by mixing 10 mL of water dispersed GO solution and 15 mL of dimethyl sulfoxide (DMSO) dispersed polymeric solution, under stirring and constant temperature (60 °C) for several hours. The hybrid nanocomposite was dropped on top of the sensing cavity space of the SAW sensor and let dry for 10 min in a hot environment (50 °C).

**Figure 2:**
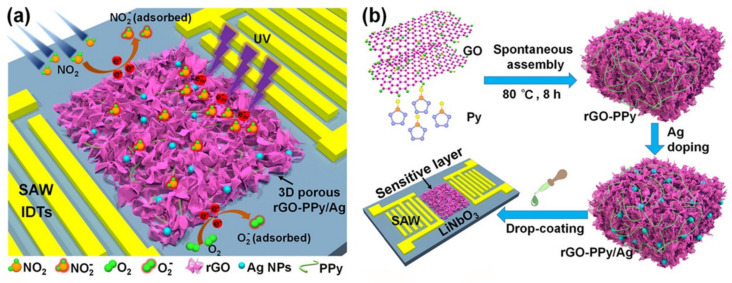
(**a**) Schematic illustration of the adsorption mechanism of NO_2_ molecules on the proposed SAW sensitive layer. (**b**) Synthesis process of 3D porous architecture rGO−PPy/Ag composite. Reprinted with permission from [37]. Copyright 2021 American Chemical Society.

PPy is an organic conjugated CP, benefiting from increased mechanical stability, very good electrical properties and low toxicity [40]. The electrochemical synthesis of PPy is facile and can be performed at neutral pH [41], on many different substrates, making PPy an intensively studied CP. An interesting aspect is that pyrrole (Py) can reduce GO to rGO [42] and the oligo-Py are adsorbed under thermal treatment by π-π stacking between GR sheets. Moreover, the combination of rGO-PPy was reported to be able to form aerogels [43], at room temperature. For this reason, GO was mixed with pyrrole to form a porous aerogel PPy-rGO, that was further functionalized with Ag nanoparticles (NPs) (Figure 2b) to increase the reversibility and room-temperature performances of the SAW device [37]. The water-based GO dispersion was mixed, under stirring, in heated media, with the monomer pyrrole (Py) to form a porous hydrogel. The porosity of the hybrid nanomaterial is a benefit due to the existence of adsorption sites for gas diffusion. The PPy-rGO was deposited on the sensing area of the SAW device, between the two IDTs. When NO_2_ molecule reach the surface area they turn to NO^2−^, while O_2_ turns to O^2−^. (Figure 2a). The hybrid aerogel was obtained via a supercritical drying method. This method required no other reagents, due to the redox interaction between the GO and Py, where GO reduces to rGO, while Py polymerases to PolyPy.

The concept of carbon nanotubes (CNT) was introduced for the first time by Iijima, in 1991 [28], and since then it showed great interest in a tremendous amount of applications. The synthesis routes for CNTs with different yields involve several methods, such as arc discharge, which was intensively studied by the group of Iijima [44,45,46], chemical vapor deposition (CVD) [47,48,49], plasma torch [50,51], liquid electrolysis [52,53,54] and laser ablation [55,56]. The two forms of CNT are single-walled carbon nanotubes (SWCNTs) which involve one enfolded sheet of GR and multi-walled carbon nanotubes (MWCNTs) which consist of several SWCNTs of smaller and smaller diameter, one inside the other [57]. SWCNTs can be synthesized in three forms: chiral, zigzag and armchair [58], and in regard to these shapes, SWCNTs present either semiconducting or metallic electrical properties. The free electrons travel within the entire length of the carbon tube at a high speed, due to its one-dimensional structure. Amongst the advantages of SWCNTs we can report excellent mechanical strength (higher than diamond) and thermal conductivity. Also, the literature describes the superconductive behavior of SWCNTs at low temperatures [59]. Usually, the inner diameter of SWCNTs is between a few nm and a few tens of nm.

For NO_2_ sensing, semiconducting SWCNTs based transistors were reported, in 2000, where CNT was obtained via the CVD method on Si substrate [60]. Poly (m-aminobenzene sulfonic acid) was added to the matrix of SWCNTs [61] for an increased sensitivity toward NO_2_ detection and better processing ability. Though, the operational temperature was still high, in the range of 270–570 °C.

For room temperature functionality SWCNTs were deposited over a SAW sensing area, using the Langmuir–Blodgett (LB) technique [62]. To be used in the LB trough, the SWCNTs (commercially available) were filled in a cadmium arachidate (CdA) matrix. The inner diameter of the SWCNTs was on average 1–5 nm, with a range of 1–10 µm in length. To obtain the SWCNTs-CdA nanocomposite, the recipe started by mixing arachidic acid solution (in chloroform) and SWCNTs dispersion (in chloroform) and then stirring, and ultrasonicated for 1 h. A very small volume of this mixture was added to an aqueous 0.1 mM CdCl_2_ solution. This nanocomposite was deposited on the active area of the SAW device in 10 to 30 layers (each layer with a dipping layer of 14 mm/min) with the thickness of 28 nm and 84 nm respectively.

MWCNTs are formed from several individual one-sheet graphene cylinders which form a weak bond in between. In comparison to SWCNTs, they can be synthesized with higher purity and fewer defects, also, their bulk synthesis is easier [57]. In 2008, a comparison between SWCNTs and MWCNTs was performed for NO_2_ detection [63]. The results showed the best sensor response for SWCNTs was at 150 °C, and for MWCNTs at 200 °C. The sensors sensitivity was higher for the SWCNTs, meaning that individually packed cylinders of CNT have better performance, to form the gas sensing layer.

For room temperature functionality and increased sensitivity, MWCNTs were doped with copper phthalocyanine (CuPc) and tested for NO_2_ detection [64]. CuPc is a metallophthalocyanine, broadly explored as a synthetic die, but for this application, it is used as a hole-transporting material. For the formation of the hybrid material, a few mg of CuPc and carboxylated MWCNTs were added together, dispersed in chloroform and ultrasonicated for 2 h. For the preparation of the CuPC/ZnO-MWCNTs, a few mg of nano ZnO were mixed with the as-prepared CuPC-MWCNTs and the same steps, detailed above, were followed. The obtained hybrid suspensions were individually sprayed on top of the SAW device using a nitrogen flow, and dried for half-hour at 150 °C. The drying step allows the elimination of the organic solvent and increases the adhesion of the material to the substrate. The deposited hybrid fibers are arranged like a net on top of the sensing area of the SAW device.

### 2.2. Metal Oxide Semiconductors (MOS) for NO_2_ Sensing

Amongst the sensitive materials employed for NO_2_ sensing, MOS have drawn an increased consideration in the last decades. They signify the most explored group of materials for gas sensing, mostly because nowadays the sizes of MOS nanoparticles are between 1–100 nm [65] and the benefit of these ultra-small sizes is the formation of a great number of active sites. The transport of holes and electrons, in nanosized MOS materials is highly influenced by morphological properties, such as size and geometry. With the decrease in size, the specific surface area increases and also certain geometries impact differently the number of active sites. For instance, a cubic shape, in comparison with sphere, tubes or rod shapes, displays facets and due to this, a larger number of active sites are available on the surface of the nanomaterial.

The adsorption and desorption of NO_2_ molecules at the surface of the sensing layer generate changes in the electrical conductivity of the MOS material. Therefore, besides the morphological properties of the nanomaterial, the chemical and electronic properties, such as the position of the Fermi level, the bad gap, and the network connection of the crystallites [66] are also extremely important for developing sensitive NO_2_ sensors.

MOS nanomaterials present several advantages such as: simplicity of use, ease of fabrication, low costs, compact size and simple sensing mechanism [67]. They can be divided into two types in relation to the charge carrier: n-type and p-type. The difference between them is that for n-type the charge carriers are the electrons and holes are for p-type. Nevertheless, they require high-temperature to operate (100–600 °C), and high energy depletion, shortening the life of the sensors and limiting their ability to be used in real-life applications as on-site portable devices. Moreover, their stability over time is reduced because the film layer is affected by the high operating temperature and the sensibility decreases, as well. For NO_2_ sensing the most used MOS materials are: tungsten trioxide (WO_3_), Lead Zirconium Titanate (PZT), Indium Tin Oxide (ITO), and titanium dioxide (TiO_2_).

In the past years, typical 2D WO_3_ structures such as thin films, nanosheets, or nanoplates have also drawn considerable attention in the gas-sensing field due to the high surface-to-volume ratio and modulated surface activities, surface polarization, and rich oxygen vacancies. Because the gas reaction is a surface phenomenon, the thin films are very suitable for gas sensors. Especially the gas sensing performances will be greatly enhanced if the thin films are composed of porous sub-microstructure [68].

WO_3_ nanocomposite material was used in 2010 [11] for NO_2_ detection at room temperature. While the WO_3_ thin film showed great performance for gas detection at high temperatures, the presence of PANI decreases the sensor’s functioning temperature, up to 24 °C. PANI is another intensively considered conductive polymer, representing the polymeric state of aniline. It can occur in many oxidation states, and therefore, in many shapes [69]. PANI is a polymer that can be obtained very simply [70], which owes high stability in the environment [71], making it the perfect candidate for developing sensors that will be used on-site. This type of organic polymer has metallic features such as electrical, optical and magnetic, but it still holds its structural polymeric comportment. PANI consists of conjugated double bonds that confer a high electrical conductivity, and it does not require any other conductive type of material. Nevertheless, the addition of a dopant in the PANI backbone increases the conductivity of the material by forming a high conductive nanocomposite. A PANI doped with WO_3_ nanocomposite was formed using a complex and time-consuming synthesis route. Briefly, it started by mixing WCl_6_, which is the most common reagent used in the preparation of several W compounds, with propanol in the ice bath. NH_4_OH was added to complete the hydrolysis process and was left overnight. To convert the obtained precipitate into a colloid suspension, a three-day reflux step was involved. Aniline dissolved in HCl was dispersed in the gel-type WO_3_ suspension. To start the polymerization process, (NH_4_)_2_S_2_O_8_ was added to the system dropwise for 20 h. Finally, the PANI-WO3 nanocomposite was obtained and after cleansing it was diluted in propanol and further used to coat the sensing area of a SAW device developed for NO_2_ sensing.

ITO is an n-type MOS nanomaterial that benefits from transparency and high conductivity, acting as a perfect candidate for the development of several applications such as: solar cells or thin-film transistors [72,73]. This oxide was used as a sensitive thin film for NO_2_ detection [74] using a SAW sensor, benefiting from increased electrical properties than the single oxides, indium oxide and tin oxide own separately. The deposition of ITO is mainly achieved by spray pyrolysis or RF sputtering, but these methods waste a large amount of indium. A better choice was to print an ITO nanoparticle-dispersed ink directly on the device [75].

ITO is not a cheap material, because the indium precursors are expensive, and much more because it is not widely spread on earth [76]. For this reason, a replacement for indium is necessary, or at least a different method to be developed for ITO manufacturing, one that would use less amount of precursor. Since chemical routes have also been intensively studied for ITO synthesis, Lim et al. [74] described a single-step solvothermal method for ITO nanoparticles synthesis. The solvent used for the reaction was surfactant-free methanol alcohol and at 250 °C temperature, a cubic shape and 20-nm size was obtained. No pre- or -post-treatment was performed.

ZnO was reported to be the most sensitive and selective nanomaterial for NO_2_ gas detection [77]. ZnO is one of the most common MOS materials, and exists mostly in three crystalline forms: cubic zincblende, hexagonal wurtzite and simple wurtzite. In the wurtzite type of ZnO, the most observed defects are the oxygen vacancies. In 2008, An and coworkers [78] investigated the adsorption of NO_2_ on the surface of 1D defect-free ZnO nanoparticles, and also on ZnO nanoparticles that presented oxygen vacancies. On the nanomaterial that showed defect sites, the gas molecules were bound 3 times stronger.

One way to improve the electronic performances of ZnO for NO_2_ sensing is the surface functionalization with different optical-active materials such as: metal nanoparticles [79,80], semiconducting materials [81,82] or dyes [83,84]. In this way, the number of free electrons in ZnO increase under light illumination through photo-induced charge separation and transfer processes [85].

In 2017, Rana and coworkers [86] described ZnO as metal oxide used for NO_2_ selective and sensitive detection. From the family of ceramic materials, PZT is one of the perovskites intensively studied as a piezoelectric substrate for developing SAW sensors. PZT brings benefits such as chemical stability, physical strength, and low-cost manufacturing. In 2018, the same group [87] proposed PZT to replace the ZnO thin film for NO_2_ gas detection, because PZT is also able to enhance the coupling coefficient of the SAW device. The 100 nm PZT layer was obtained via pulsed laser deposition (PLD) technique.

A nanohybrid material is generally a combination of at least two nanosized materials and their biggest advantage is that they benefit from the properties of each individual nanomaterial. For gas sensing, the operation temperature is diminished to room temperature when a nanohybrid is used as a sensing layer in a SAW device.

Graphitic carbon nitride (g-C_3_N_4_) is an attractive conjugated 2D polymer with a layered graphite-like structure, having a basic sheet formed from triazine subunits linked through N– groups. Besides chemical and thermal stability, the main benefit of this nanomaterial is represented by the small bandgap ~2.7 eV [88,89], which enhances also the electrical conductivity.

Titanium dioxide (TiO_2_) is one of the n-type MOS materials which has a nanostructure similar to nanoplatelets (NP). This MOS material has several advantages such as cost-effectiveness, eco-friendly and nontoxic nature, presenting high stability and a bandgap of ~3.1 eV [20,21,22]. Besides, the surface defects existing on the TiO_2_ facet (such as limp bonds and unsaturated coordinated atoms) provide more active O_2_ absorption sites for the effective NO_2_ gas sensing performances [90].

In 2022, Pasupuleti [13] integrated g-C_3_N_4_ with TiO_2_ NPs, to form a nanohybrid composite allowing NO_2_ gas molecules to be absorbed. The synthesis of g-C_3_N_4_ was reproduced from [27] and consisted of a thermal polymerization recipe starting from 10 g of melamine that was calcinated for 2 h at 600 °C to obtain a yellow fine powder. The white precipitate of TiO_2_ was synthesized via a hydrothermal method starting from tetra butyl titanate. The two nanomaterials were mixed in a 3:1 ratio (g-C_3_N_4_:TiO_2_) and dispersed in an EtOH solution. The nanohybrid dispersion was drop-casted on the cavity sensing area of the SAW device.

### 2.3. Quantum Dots (QDs) for NO_2_ Sensing

Due to tremendous interest in the manufacturing of gas sensors, semiconductor nanomaterials that have a quantum confinement effect, named quantum dots (QDs) were included in several research studies [91,92,93,94]. Only limited quantum dots materials reported in the literature [95] show a continuous evolution of the quantum confinement effect from 1D to 0D system. For NO_2_ sensing, the inability to operate at room temperature of MOS-based sensors was reduced by adding QDs in the MOS lattice.

Until now, only a few colloidal QDs have been integrated with SAW devices for NO_2_ sensing, such as lead sulfide (PbS) QDs [90,92], and tin sulfide (SnS) QDs [93], and bismuth sulfide (Bi_2_S_3_) QDs [94].

The bulk state of the semiconductor PbS presents a slim band gap of 0.4 eV [96]. The PbS QDs benefit from excellent opto-electronic features, being an interesting choice for a variety of applications, such as photodetector/photocatalysis or solar cells [97,98,99].

A complex synthesis method based on a cation exchange was used in [92] to obtain PbS from CdS colloidal QDs and introduced as a selective layer for NO_2_ detection. The authors synthesized Pb, Cd and S precursors prior to the formation of the colloidal-dots dispersions. The Cd precursor was formed by mixing 1-octodecene (ODE) with oleic acid (OA) and CdO for almost half an hour at a high temperature of 250 °C, and cooled down to 24 °C. The S precursor was obtained by mixing oleylamine (OLA) with a very small volume of aqueous ammonium sulfide solution. The CdS QDs were formed at room temperature by combining the two precursors. The synthesis of the Pb precursor started by injecting PbCl_2_ in OLA solution at 140 °C for a half hour and cooled down in a water bath. The Pb precursor was mixed with the CdS QDs to obtain the PbS QDs, which were rinsed with EtOH and hexane, and finally dispersed in octane. The PbS QDs was coated on top of the sensitive area of the SAW sensor and treated with Pb (NO_3_)_2_ to eliminate both OA and OLA from the surface, which would have acted as insulating layers, limiting the gas adsorption. This treatment created cracks in the film, becoming porous and enhancing the ability of the material to adsorb NO_2_ molecules.

In 2019, Li and his group [90] used the same cation exchange route described above to synthesize PbS QDs as NO_2_ sensitive layer, starting from CdS QDs. First, the authors synthesized Cd-oleate to use it as a Cd precursor, by heating a mixture of CdO, OA, and ODE up to 260 °C for several minutes. The S precursor was synthesized by dispersing (NH_4_)_2_S in a few mL of OLA, followed by adding it to the formed Cd-oleate and stirring for 1 h, at room temperature. The obtained CdS was dispersed in toluene. The Pb precursor was formed by mixing PbCl_2_ in OLA while heating it for half-hour up to 140 °C. Then CdS dots dispersed in ODE were injected into the flask containing the Pb precursor and cooled down in a water bath. Hexane and OA were added subsequently at different temperature values and the final product was washed and dispersed in octane, denoted PbS QDs. The authors used the same treatment, described above, to create the porosity in the film and the reaction mechanism is basically identical.

Tin sulfide (SnS) belongs to the family of transition metal chalcogenides (TMCs) and it has been employed in gas sensing applications due to its great response at room temperature [100]. The presence of surplus S atoms in the structure of SnS confers the p-type behavior to the material, though some researchers studied the n-type of SnS induced by the S vacancies [101]. Both types have been studied for gas sensing, and while the p-type has prominent high resistance, the n-type shows low resistance [102], promoting the development of flexible gas sensors [103].

The same group [93] selected SnS QDs as coating sensitive layer for the SAW device, due to the fact that its toxicity is lower than PbS QDs. So, SnS QDs were reported for the first time as sensitive material for NO_2_. The SnS-based solution was prepared using a hot-injection technique, starting by mixing SnCl_2_, OA, 1-octadecene (ODE) and trioctylphosphine (TOP) and heating the flask at 60 °C, under a vacuum. After increasing the temperature to 100 °C, thioacetamide (TAA) and OLA were rapidly injected into the flask. The heating process was stopped suddenly after injection and the obtained product was cleansed in EtOH and dispersed in octane. The colloidal dispersion consisting of spherical nanoparticles of 5 nm average diameter was spin-coated on the sensitive area of a SAW device to form a thin film of SnS.

The chalcogenides that use Bi as a metal element are attractive n-type MOS materials used intensively for gas sensing applications [104,105,106]. Bismuth trisulfide (Bi_2_S_3_) is a 1D nanostructured semiconductor that owns an orthorhombic structure with a narrow bandgap of 1.3 eV [107,108]. It can be synthesized in different shapes [109,110,111,112], such as: nanotubes, nanorods, nanobelts, and nanowires. The different physical nanosized aspect creates unique electronic, optical and mechanical properties of Bi_2_S_3_ QDs creating perfect opportunities to be integrated into nanoscale electronic devices [107].

In 2021, Bi_2_S_3_ QDs [94] were investigated as a sensing layer for NO_2_ detection using a SAW device described in the literature [90,93]. The nanobelts of Bi_2_S_3_ were synthesized using a solvothermal method, starting from Bi(C_6_H_5_)_3_ as Bi precursor and Bis(phenylmethyl) disulfide as S precursor. Both substances were prepared in oleylamine (OLA) and mixed individually with ethanol dispersed polyvinyl pyrrolidone (EtOH-PVP). Both solutions were combined in an autoclave and heated at 180 °C for several hours. The obtained precipitate was dispersed in EtOH and used like this to coat the SAW active area.

## 3. SAW Sensors for NO_2_ (Gas) Detection

### 3.1. Interdigital Transducers and SAW Sensors Configurations

Interdigital Transducers (IDTs) are key elements in the design of the SAW (gas) sensors. These periodic metallic electrodes, also known as fingers, deposited on piezoelectric substrates have a double function: to convert the electrical radio frequency (RF) signal into a surface acoustic wave by generating mechanical forces and vice versa. The IDTs can have several configurations, such as bi-directional electrodes, split electrodes, Single Phase Unidirectional Transducer (SPUDT) electrode configuration, etc. [17]. The performances of the NO_2_ SAW sensors strongly depend on the IDTs configurations, and a number of fingers, but at the same time on the properties of the piezoelectric substrate or the coating material. To generate the acoustic waves and to detect changes in their characteristics, two NO_2_ SAW configurations are mainly used: surface delay lines (SDL) and surface acoustic wave resonators (SAWR).

**Figure 3:**
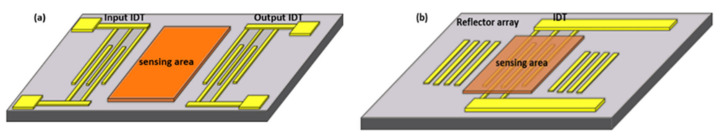
The schematic diagram and the design parameters of (**a**) an SDL and (**b**) one port SAWR.

Typical SDLs comprise two IDTs (input and output transducers) placed at a certain distance from each other. The separation area is usually coated with the gas-adsorption material which produces a time delay between the signals of the two IDTs, depending on its interaction with the surface acoustic wave, which alters its propagation speed, and the length of the transit zone. The schematic diagram and the design parameters of an SDL can be seen in Figure 3a.

The resonator’s configuration consists of two IDTs for the generation and detection of the surface acoustic wave and a pair of grating reflectors placed near the outer edge of each IDT, which creates a resonating cavity between IDTs. Because this configuration implies two IDTs, the design is called a two-port resonator. If the resonator’s configuration used only one IDT for both the transmission and reception of the signal, the design is called a one-port resonator (Figure 3b). In the resonator’s configuration, the sensing material can be directly deposited over the IDTs [16].

### 3.2. SAW Sensor Test Methods for Gas Detection

**Figure 4:**
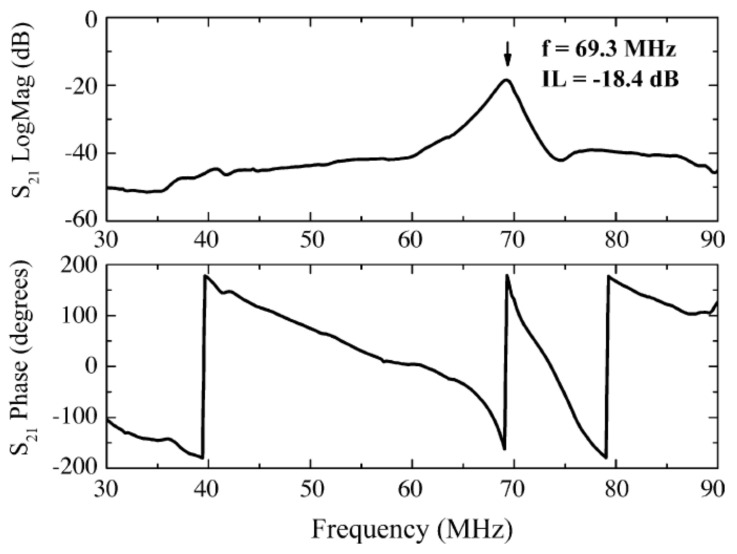
Typical S_21_ transmission response (magnitude and phase of a layered ZnO/LiTaO_3_ SAW two−port resonator with 10 monolayers LB 75 wt.% SWCNTs−in−CdA nanocomposite. Reprinted with permission from Ref. [62]. Copyright 2007 Elsevier.

The choice of techniques and equipment used for the characterization of the SAW-based sensors largely depends on the type of features to be analyzed. The Vector Network Analyser (VNA) is one of the most commonly used measuring equipment [13,31,37,62,86,87,90,92,93,94] due to its advantages: extremely wide bandwidth, excellent frequency stability, outstanding accuracy of amplitude and phase measurements. The VNA measures complex parameters of single port devices, i.e., the reflection coefficients, or full scattering matrix [S] of two-port transmission type circuits. At the same time, the reference planes of all measured parameters can be translated to the sensor terminals if appropriate calibration kits are used [19]. Typically, the sensor’s transfer characteristic |S_21_| modulus shows a fairly pronounced maximum, sometimes called resonant center frequency (RCF). It is, therefore, preferable to determine this specific frequency due to its dependence on the concentration of the analyte for which the sensor was designed. If the transfer characteristic does not show a clear maximum, but has an almost flat shape around RCF or it has (multiple) fluctuations, the RCF reading becomes uncertain. Therefore, the actual RCF position can be approximated using certain symmetric analytic functions; as an example, Pasupuleti et al. [31] used Gaussian curve fitting to find out the best estimation of the RCF value. Under these conditions, an alternative solution is to observe the phase variation of the transfer coefficient, since it is a measurable quantity even in situations where the |S_21_| modulus is almost constant in the frequency band of interest. This specific behavior is well illustrated in Figure 4, which shows the amplitude and phase variation of the broadband transfer characteristic for the resonant sensor described in [62].

With all the advantages mentioned, VNA is an expensive equipment, while its intrinsic accuracy is only assured under controlled environmental conditions, which excludes its use outdoors. It is therefore necessary to keep the sensors and their measurement enclosures at controlled test conditions, as any variation in ambient temperature can introduce unacceptable measurement errors, especially if piezoelectric substrates with a high coefficient of thermal expansion are used.

A solution used by many authors [11,33,64] to reduce these costs is based on inserting an SDL or SAWR sensor into the positive feedback loop of a high-gain amplifier. Therefore, the whole circuit becomes a radiofrequency (RF) oscillator whose operating frequency is determined by the complex transfer parameter S_21_ of the sensors and, in particular, by the phase shift of the acoustic wave passing through the transit zone. However, the frequency of this RF signal depends not only on changing the physical and chemical response of the sensing layer, but also on so-called common-mode environmental factors, such as temperature, pressure and relative humidity. To significantly reduce the influence of these disturbing factors, differential structures are used, which consist of two sensors of the same type, but where only one of them has a sensitive layer on the signal transit area. In this case, the frequencies of the two oscillators can be measured separately with the same frequency meter, and the difference between the frequency values, calculated later, is proportional to the concentration of the studied analyte. Also, the signals from the outputs of the two oscillators can be fed into a double-balanced mixer, so that it will only be necessary to measure the intermediate frequency, of lower value, at the mixer output.

The two categories of gas sensor characterization methods described above are based on the use of high-performance, and therefore expensive, equipment that can be used mainly under laboratory conditions. The presence of toxic gases in the environment requires in situ monitoring of their concentrations, with simple, but sufficiently precise means of measurement and specific procedures. In this context, in [74] the authors presented a circuit solution currently used for wireless temperature measurement of vehicle wheels or tires [113]. This was adapted to add the measurement of both NO_2_ and CO_2_ gas concentrations. To this end, the SAW structure designed for the temperature measurement was supplemented by two groups of reflector elements flanking the transit zones covered with layers specific to the detection of the two types of toxic gases. The transfer coefficients of the reflectors were selected to attenuate the acoustic wave in a controlled way, while the variation of the acoustic wave propagation time in the sensing layers due to different gas concentrations was used for measurements. Due to the 15 mm total length of the acoustic wave propagation path, the response signal duration reaches about 7 μs.

## 4. Layered SAW Resonator (SAWR) Configuration for NO_2_ Detection

**Figure 5:**
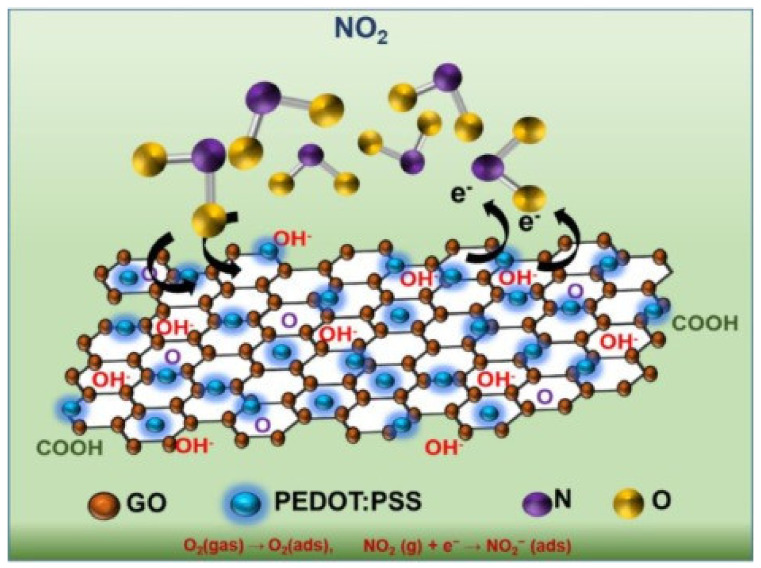
Sensing mechanism of GO−PEDOT:PSS under NO_2_ gas ambient. Reprinted with permission from Ref. [31]. Copyright 2021 Elsevier.

In 2021, Pasupuleti et al. [31] reported a SAW sensor for NO_2_ detection fabricated on langasite (LGS) piezoelectric substrate. The developed device configuration was based on a two-port SAWR. The layout of the sensor consisted of two IDTs, two pairs of reflector gratings electrodes to form a standing wave pattern and a center sensing area placed in between the input and output IDTs. Each IDT had 100 finger pairs, with λ/4 = 5 µm finger width and 20 µm electrode periodicity. The two periodic reflector gratings had 150 electrodes, with an 800 µm aperture height. The sensing area of the SAWR was 5 mm. For this configuration, the developed device showed a resonant center frequency of 136.1 MHz, and 20.16 dB signal attenuation. The manufacturing process of the sensor was simple, using a single photolithography mask. To perform the pattern transfer of the sensor from the mask onto the LGS material, a negative photoresist was first spin-coated onto the piezoelectric substrate and patterned by photolithography. A thin film of Ti/Au of 10 nm/50 nm was subsequently deposited via e-beam evaporation. The device configuration was finally obtained using the lift-off process. The sensing area of the SAWR was subsequently coated with a GO-PEDOT-PSS nanocomposite acting as a chemical interface for effective NO_2_ detection, leading to a resonant frequency drop to 135.78 MHz and about 29.78 dB transmission attenuation (measured in transmission mode with a VNA). In terms of sensing action, GO chemically bonds to the polymeric mixture through π-π * interactions, which are intercalating between GO’s crystal lattice and polymeric membrane. Each material taken individually has poorer sensitivity towards NO_2_ sensing, but together, as a nanohybrid material, the surface area increases and the size of the nanopores decreases due to the fact that the polymer was blended in the crystal lattice of the GO. When exposed to NO_2_, the gas molecules were absorbed and entered the sublayer of the nanohybrid material (Figure 5), contributing to an increase in the mass of the sensing layer. Sensitivity determination of the sensors was carried out under strict control of the relative humidity (~2.3%) and ambient temperature (25 ± 2 °C). Small variations in the amplitude of the transfer characteristic (S_21_) of the measured sensor required the use of Gaussian curve fitting to determine the value of the frequency at which the S_21_ module had its maximum value. The sensitivity of the sensor, defined by the ratio of relative variation Δ*f* of the operating frequency to the concentration *c* of NO_2_ (expressed in ppm) follows a parabolic law:

(1) $${\left(\Delta f\right)}^{n}=K\cdot c$$

where the exponent *n* and the constant *K* can be determined from actual experimental data. Due to the non-linear response of the sensor, an average sensitivity of +57 Hz/ppm was calculated for NO_2_ concentrations over the 0–100 ppm range. The calculated detection limit was 175 ppb.

**Figure 6:**
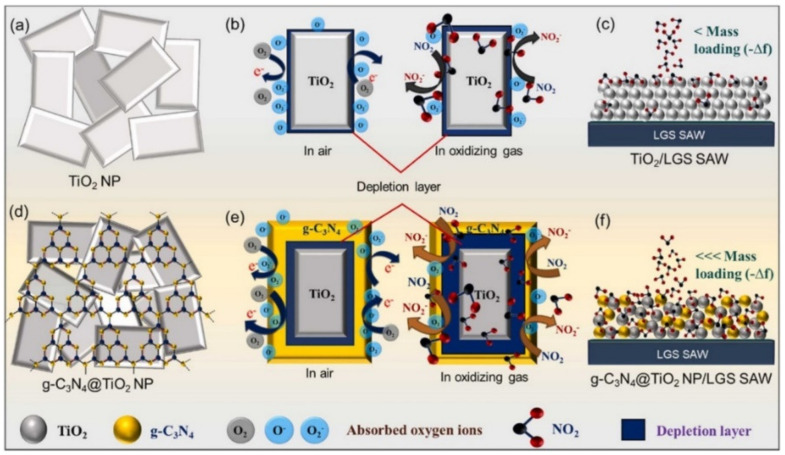
Schematic illustrations of the plausible NO_2_ gas sensing mechanism of (**a**–**c**) pristine TiO_2_ NP and (**d**–**f**) g−C_3_N_4_@TiO_2_ NP hybrid nanocomposite heterostructures at RT. Reprinted with permission from Ref. [13]. Copyright 2022 American Chemical Society.

One year later, in [13], the group used the same LGS SAWR designed to detect the NO_2_ gas. They used a 2D g-C_3_N_4_@TiO_2_ hybrid nanocomposite as sensitive material, instead of GO-PEDOT:PSS, to be effective for room temperature NO_2_ detection. By loading TiO_2_ on g-C_3_N_4_ the nanohybrid material (Figure 6) benefits from a large surface area due to the mesoporous nature of the sample, which allows the transportation of a large number of gas molecules and penetration into the interfacial layers of the TiO_2_@g-C_3_N_4_. Besides this aspect, the high adsorption abilities for NO_2_ were attributed to the presence of the hydroxyl and amine functional groups, defective O_2_ vacancies, the depletion layer and also to the 001 orientation of the NPs structures. Furthermore, the synergetic coupling between the two nanomaterials is achieved by the formation of an n-n heterojunction improving the charge carrier density through π-π * bonding. This event provides more active sites and increases the specific surface area for the adsorption of NO_2_ gas molecules.

These modifications in the chemical composition of the sensing layer produced a variation in both the resonant frequency and signal attenuation through the SAW devices. Compared to the values of 136.1 MHz and 19.9 dB obtained for pristine LGS-SAW, the use of TiO_2_ NP led to a decrease of the resonant frequency to 135.85 MHz and the attenuation became 24.2 dB. Then, loading TiO_2_ on g-C3N4 the nanohybrid material, 135.73 MHz resonant frequency, and 29.3 dB attenuation were obtained. This approach had a positive effect on some sensor’s characteristics, namely, their sensitivity increased from −85 Hz/ppm up to −197 Hz/ppm, due to successive changes done in the composition of the sensing layer. The response of this sensor model remained parabolic, but with different values of exponent *n* and constant *K*, compared to [31]. A favorable effect on the sensitivity of the sensors was the increase in ambient humidity.

A new sensor based on a two-port SAW resonator designed with a ZnO sensing layer for NO_2_ gas detection was presented in [86]. The layout of the SAW device consisted of a pair of IDs, each of them having 110 finger pairs, with a 7.895 µm finger width and acoustic wavelength of 31.58 µm. Two periodic reflector gratings with 180 shorted fingers were placed at the edge of IDTs. By using this configuration, the operating frequency of the SAW resonator was 99.5 MHz. The manufacturing process of the SAW device started with the deposition via e-beam evaporation of an Al film, with a thickness of 200 nm, over the ST-cut quartz piezoelectric substrate. The IDTs pattern transfer was performed by the photolithography technique. A c-axis oriented ZnO thin film, with a thickness of 100 nm was then deposited over the fabricated SAW devices via RF magnetron sputtering technique and patterned by photolithography (in the electrical pad areas), thus resulting in a slight reduction of the resonance frequency, down to 99.477 MHz. The ultra-small size of ZnO crystallites (having an estimated average size of 24 nm for each crystallite) brings the advantage of increased active sites, that allow NO_2_ molecules to be adsorbed on the surface. The transfer characteristic of this two-port sensor was measured with a VNA for several values of NO_2_ concentration. Both the variation of the frequency at which the amplitude of the sensor output signal showed a maximum, and quite significant change in the amplitude of this signal were observed. According to the authors, the sensitivity of the device determined for a NO_2_ concentration of 16 ppm showed a resonant frequency deviation of 112 kHz. The variation of frequency with concentration was non-linear, with a smaller slope for minimum and maximum concentrations, and more pronounced at concentrations within 8–12 ppm range. The selectivity of the sensor was also determined by successively exposing it to several possible interfering compounds, each of them having the same concentration of 16 ppm: liquefied petroleum gas, methane, hydrogen, NO_2_, CO, and acetone vapors. It was also observed that the measured resonance frequency deviation for NO_2_ was about an order of magnitude higher than for any of the other analytes.

One year later, the same group published a new article presenting an efficient surface acoustic wave-based wireless NO_2_ gas sensor [87]. The authors used the same sensor design as presented in [86], but instead of the ZnO sensing layer for NO_2_ detection, they chose PZT. Therefore, after the Al configuration of the IDs, a resist masking layer coated the contact pads of the structure and an amorphous PZT thin film of 100 nm thickness was deposited using the pulsed laser deposition (PLD) technique. At the end of the fabrication process, the resist was removed along with the PZT on its surface facilitating the wire bonding for electrical contacts. The measured resonant frequency of the fabricated devices without PZT was 99.4 MHz. Since the acoustic velocity of PZT is lower than the ST-quartz, a slightly shifted frequency value up to 99.193 MHz was observed for PZT-coated SAW devices. The PZT film of 100 nm thickness, deposited on the sensing area of the SAW device showed a disordered structure, a rough surface morphology that assures a high number of active sites accommodating a large amount of NO_2_ molecules. The transfer characteristics of these two-port sensors were determined by immersing them in an environment in which the NO_2_ concentration varied between 80 ppm and 250 ppm for calibration curve plotting. The resonant frequency change of the device in the enclosure was measured using a VNA, obtaining the linear approximation of its characteristic with a slope of approximately −9.6 Hz/ppm.

In 2016, Hu et al. [64] developed in their work an NO_2_ sensor based on nanocomposite material-based on MWCNTs. The SAW resonator used in this paper was a commercial one (ACTQ433). The structure fabricated onto quartz substrate comprised two Al-metalized IDTs, with 0.5 µm fingers periodicity, and a pair of reflecting gratings. The center frequency obtained for this configuration was 433.92 MHz, with a variation of ±75 MHz for different devices. MWCNTs doped with copper phthalocyanine (CuPc) were deposited on top of the IDTs for NO_2_ detection. Additionally, a hybrid suspension containing a few mg of nano ZnO mixed with the CuPC-MWCNTs was tested as a sensing material for the NO_2_ absorption. The net-like arrangement of the CuPC-MWCNTs and CuPC/ZnO-MWCNTs hybrid fibers provides a porous morphology, that promotes the adsorption of NO_2_ molecules into the material. The authors used a NO_2_ concentration measurement system consisting of two Pierce oscillators based on two-port SAW resonators whose output signals were fed into a broadband double-balanced mixer. Since only one transducer had the sensing area covered with CuPC-MWCNTs and CuPC/ZnO-MWCNTs, the IF (intermediate frequency) signal frequency became proportional to the NO_2_ concentration in the measuring gas chamber. The frequency difference between the oscillators ranged from 500 Hz (at 0 ppm NO_2_) to almost 5 kHz at 100 ppm NO_2_. Since the response was linear only up to 60 ppm or less, after which saturation was observed, the average sensitivity of the system was 4836 Hz for the whole 0 ppm to 100 ppm range. All measurements were performed at +50 °C. The 90% response time of the sensor was estimated to be 500 s, but the 90% recovery time was longer, at about 3000 s.

**Figure 7:**
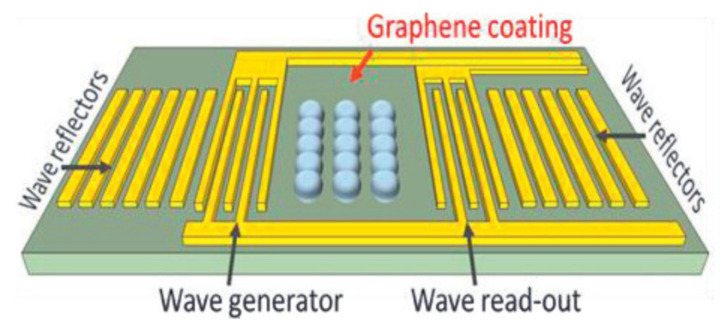
The designed two-port SAW sensor in a dual configuration. Reprinted with permission from Ref. [33]. Copyright 2014 Elsevier.

Another graphene-coated Rayleigh SAWR for NO_2_ detection was presented in [33]. In this paper, the acoustic device was manufactured on ST- cut quartz piezoelectric substrate due to its high-temperature stability. The proposed two-port resonant device (Figure 7) operates in a dual configuration removing the common mode interferences on the sensing signal such as variations in ambient temperature or pressure. This layout allowed one part of the dual device to be used as the reference channel, while the other side acted as a sensing region. Moreover, the dual arrangement reduced the noise level and maintained a high signal to noise ratio.

Each IDT comprised a set of 60.5 finger pairs, with 3 µm finger width and 720 µm acoustic aperture. The IDTs were surrounded by 600 reflector gratings electrodes with a pitch of 6 µm. The distance between the input and output IDTs was set at 303 µm, to create a standing wave pattern. The developed sensors had a resonant frequency of 262 MHz. UV lithography was used for the fabrication process of the sensors and aluminum was chosen as the metallization material [114]. GR was deposited on one side of each dual device and used as the sensing material. The binding of NO_2_ molecules to the GR ink is enhanced by the presence of defects in the exfoliated GO lattice, showing an increased sensitivity of the developed SAWR sensor. The measurement system described in this paper consisted of two SAW sensors, of which only the measuring one was coated with a sensing layer. Each of these two-port sensors was inserted into the feedback loop of a high-gain amplifier to form an oscillator with an operating frequency of about 262 MHz. Thanks to this arrangement, the operating frequencies of both oscillators were measured with a frequency meter, which is much cheaper than the VNA, being preferred by other authors. A sensitivity of about 25 Hz/ppm NO_2_ and 35 s response time vs 10 s recovery time were obtained.

Shen et al., reported in [11] a SAW sensor based on PANI-WO_3_ nanocomposite for the detection of nitrogen dioxide. The sensor layout was based on a two-port resonator configuration. Each IDT had 104 finger pairs, with a periodicity of 32 µm and 960 µm aperture. The sensing surface was 605 µm and each IDT was bordered by 150 strip gratings. For this specific design, the measured central frequency of the developed SAW device was 98.47 MHz. The sensor was manufactured on an ST-cut quartz piezoelectric substrate. The pattern transfer was performed by the lift-off method and aluminum with a thickness of 300 nm was chosen as the metallization material. Polyimide was then deposited over the IDTs and reflectors in order to provide the electrodes’ protection. A PANI-WO_3_ nanocomposite material was deposited in the sensing area (placed between the IDTs) of the sensor. While the WO_3_ thin film showed great performances for NO_2_ gas detection, the presence of PANI decreases the sensor’s functioning temperature. The porosity created in the nanomaterial with the polymerization of aniline, brings great advantage to the sensing system, by increasing surface area, which allows more and more NO_2_ gas molecules to be adsorbed on the surface of the SAW sensor. The operation of the differential type measurement system was based on the use of two oscillators in which SAW sensors were embedded, one of which was provided with a sensing layer and the second one was not. The oscillation frequency was f_0_ = 98.47 MHz in the absence of NO_2_, as measured with a frequency meter. The sensitivity of the measurement system was calculated using measurements for two NO_2_ concentrations (39 ppm and 77 ppm), resulting in a value of −1.397 Hz/ppm. The response and recovery times of the device provided with sensing layer were approximately equal (around 120 s each).

Penza et al. presented in 2007 a layered SAW NO_2_ gas sensor based on SWCNTs nanocomposite [62]. In this work, the authors used a two-port resonator configuration, where the IDTs comprised 38 finger pairs in each port, with λ = 40 µm. The resonant cavity of the sensor was created by using 160 acoustic reflectors placed at the edge of each IDT. The distance between IDTs was 48 λ, with an aperture of 700 µm. The manufacturing process started with the deposition and patterning of the Ti/Au (20 nm/80 nm) electrodes over the 36° Y-cut X-propagation LiTaO_3_ piezoelectric substrate. Subsequently, a ZnO guiding layer of 1.2 µm was sputtered over the structure (except in the pads area) in order to improve the electromechanical coupling coefficient of the device. The LiTaO_3_ substrate allowed the propagation of shear waves (SH) with a phase velocity of 4150 m/s, while ZnO had a phase velocity of only 2850 m/s. These results were performed in Love-mode acoustic wave propagation. The measured operating frequency of the guided SH-SAW was 69.3 MHz. SWCNTs of different layers were subsequently deposited on the SAW sensing area, using the Langmuir–Blodgett (LB) technique. To be used in the LB trough, SWCNTs (commercially available) filled in a cadmium arachidate (CdA) matrix were used as a sensing material for the sensor. The inner diameter of the SWCNTs was on average 1–5 nm, with a range of 1–10 µm in length. The tangled structure of SWCNTs-CdA nanocomposite provided a disordered surface distribution of the carbon-based material, that enhanced the gas adsorption rate, providing a good sensitivity of the sensor. In determining the sensitivity of this sensor, the authors relied exclusively on the phase measurement of the S_21_ transfer coefficient, mandatory at the same frequency, using a VNA. Due to the low operating frequency of the sensors, the phase variations were also small, only a few tenths of a degree. For example, for the NO_2_ concentration change from 2 ppm to 8 ppm, a phase variation of about 0.17° was obtained. In contrast, the selectivity was very good for some gaseous species: the phase variation was ca. 0.35° for an increase in NH_3_ concentration from 150 ppm to 1000 ppm, and a phase variation of 0.1° was determined for 1500 ppm H_2_ concentration.

The following table (Table 2) summarizes the features and performances of the above-discussed SAW sensors for NO_2_ detection.

## 5. Layered SAW Delay Line (SDL) Configuration for NO_2_ Detection

In 2021, a new rGO-PPy/Ag-based SAW sensor with UV activation exhibiting high sensitivity, reproducibility, selectivity and fast response/recovery time was reported [37]. The NO_2_ sensor was fabricated on Y-cut 128° lithium niobate (LiNbO_3_) piezoelectric substrate. The design of the SAW device was based on delay line configuration, comprising two IDTs and a sensing area placed in between. The IDTs, with λ = 16 µm, had 60 finger pairs and an aperture of 250 λ. The distance between the IDTs was 200 λ and each IDT was surrounded by 120 reflector pairs. For this specific configuration, the center frequency of the sensor was 246.99 MHz. The fabrication process of the sensor involved a single photolithographic mask. UV photolithography, gold physical vapor deposition and lift-off processes were used in order to fabricate the SAW device. The sensing area of the sensor was subsequently coated by a GO nanomaterial that was mixed with pyrrole to form a porous aerogel PPy-rGO, and further functionalized with Ag NPs to increase the reversibility and room-temperature performances of the SAW device. The NO_2_ gas diffuses to the porous PPy-rGO hybrid nanocomposite. To help gas molecules to diffuse faster, Ag was doped in between the 3D structure of PPy-rGO. Moreover, the D stacked architecture inherited the catalytic properties of AgNPs and benefited also from the formation of a larger number of active sites, due to the small sizes of AgNPs. The change in the transfer characteristic of the sensors, both in terms of the frequency of the maximum response and the change in signal attenuation, was measured with a VNA. Good linearity of the sensor frequency response was observed, which allowed to determine its sensitivity of 0.12768 kHz/ppm NO_2_ concentration, with a limit of detection (LOD) of approximately 2.37 ppm. Also, the combination of substances used for the coating layer allowed a significant reduction of the sensor response time to less than 60 s for NO_2_ concentration change between 0 and 100 ppm.

In the same year, a new paper presenting a SAW room temperature NO_2_ sensor based on one-dimensional (1D) Bi_2_S_3_ nanobelts as the sensing materials was reported in [94]. The proposed device used a delay line configuration, containing an input IDT with 220 finger pairs, an output IDT with 95 finger pairs and a sensing area placed in between. The SAW resonant structure was fabricated by conventional photolithography technique onto ST-cut quartz piezoelectric substrate. For this specific configuration, the center frequency of the developed SAW device was 199.94 MHz, and insertion loss were −15.50 dB. Bi_2_S_3_ nanobelts were deposited on the sensing area of the sensor and the authors observed a small decrease in the center frequency (up to 199.86 MHz) and insertion loss (to −18.85 dB) due to the mass load effect and the acoustic wave scattering. The porosity of Bi_2_S_3_ nanobelts contributed to increasing the sensitivity of the NO_2_ sensing platform, due to the ability to attract larger amounts of NO_2_ molecules to the surface of the sensor, which were adsorbed on the edge of the holes. The NO_2_ concentration at which the sensor response had good linearity was from 1.2 ppm to a maximum of 10 ppm, for which the VNA measured a variation in operating frequency between 0.4 kHz (at minimum concentration) and 2 kHz for 10 ppm concentration. The slope of 177 Hz/ppm of the linear approximation of the operating frequency, which varied with NO_2_ concentration was the actual average sensitivity of the device. The authors also calculated the theoretical LOD of the sensor, which was approximately 17 ppb. The authors also obtained very good sensor selectivity towards NO_2_, when contaminating the gas chamber with H_2_, toluene, ethanol, and isopropanol.

In 2018, Fan et al. [92] reported a room temperature NO_2_ SAW sensor with PbS CQDs sensing nanomaterial for NO_2_ detection. The developed device used a delay line configuration with a single SPUDT. The input/output IDTs (with λ = 18.9 µm) had 4.1 mm and 1.3 mm respectively, an aperture of 1.576 mm, and a sensing area of 1.89 mm placed in between. The proposed SAW device was fabricated on an ST-cut quartz piezoelectric substrate using the photolithographic technique and the lift-off process. A thin film of Al with a thickness of 150 nm was patterned as the IDT electrodes. A new deposition (via magnetron sputtering) of 500 nm SiO_2_ was performed over the patterned IDTs as a passivation layer, in order to protect the electrodes from erosion. PbS colloidal QDs, prepared by cation exchanging method from CdS QDs were treated after being coated on the SAW sensitive area. A special soaking treatment using Pb(NO_3_)_2_ to exchange the long-chain surface-capping ligands of OA and OLA was applied. The aim of this process was the formation of a porous film, that promoted the adsorption of the NO_2_ molecules. The sensors were measured before and after PbS colloidal QDs deposition, which significantly reduced response and recovery times from 945 s/813 s to 50 s/60 s. A central frequency of 200 MHz with an insertion loss of −12.3 dB was measured for the uncoated devices, and a shift to 199.8 MHz and −13.2 dB in central frequency and insertion loss was found for the structures with a sensing layer. Using a SPUDT version of the SDL sensor provided an average sensitivity of 0.91 kHz/ppm, calculated for NO_2_ concentration over 1 ppm to 70 ppm range, and a LOD value close to 105 ppb.

In 2019, Li et al., developed an NO_2_ sensor based on PbS colloidal QDs sensing material ([90]), operating at room temperature. The SAW device consisting of a two-port delay line configuration was designed for the fabrication on ST-cut quartz piezoelectric substrate due to its nearly zero temperature coefficient at room temperature. The input (220 finger pairs) and output (95 finger pairs) IDTs, with a periodicity of λ = 15.8 µm and a sensing area of 3.6 mm^2^ were patterned by the photolithography technique. Aluminum with a thickness of 200 nm was selected as the metallization material for the SAW device. The measured center frequency of the fabricated devices was 200 MHz, with an insertion loss of −11.7 dB. Subsequently, a thin film of PbS colloidal QDs prepared by cation exchanging method from CdS colloidal QDs was deposited by spin coating method over the sensing area of the sensor. A new characterization was performed and the authors observed a decrease of the center frequency up to 199.9 MHz and −12.7 dB for the insertion loss. This is due to the mass (PbS CQDs) loading effect. The authors used the same treatment, described in [92], to create the porosity in the film and the reaction mechanism was basically identical. The concentration range for which a linear response was obtained did not exceed 10 ppm, after which the frequency variation was quite limited. For concentrations between 0.5 ppm and 30 ppm, a variation in operating frequency between 1 kHz and 12 kHz was measured with the VNA, so that the average sensitivity was only 373 Hz/ppm, due to saturation of the sensor response. Over the linear response range (concentration of 10 ppm or less), the sensitivity was 910 Hz/ppm, with about 32 ppb calculated LOD. The sensor response was within 1 min for either detection or recovery actions.

In 2019, Li et al., presented in [93], another NO_2_ SAW sensor based on colloidal SnS QDs. The authors used the same sensor design as presented in [90], but instead of PbS colloidal QDs sensing layer for NO_2_ detection, they chose colloidal SnS QDs due to the their low toxicity as compared to PbS colloidal QDs. The presence of nanometric-sized particles in the sensing area increased the active surface area of the device. The NO_2_ gas molecules adsorption caused a mass loading and a corresponding change in the RCF. A variation of the sensor operating frequency between 0.5 kHz and 1.8 kHz (for NO_2_ concentrations between 1 ppm and 10 ppm) was measured, while the response and recovery times were 180 s and 480 s respectively. Moreover, the sensor’s sensitivity to certain interfering analytes such as NH_3_, SO_2_, CO or H_2_ was very low”.

In 2011, Lim et al., developed a multi-gas SAW-based sensor for simultaneous detection of CO_2_ and NO_2_, integrating also a temperature sensor [74]. The system was designed and manufactured on a 41° YX LiNbO_3_ piezoelectric substrate. This material was mainly chosen due to its high SAW propagation velocity (4792 m/s) and large electromechanical coupling factor K^2^ (17.2%). The propagation velocity ensured a high sensitivity of the sensor, while the large K^2^ provided a high reflectivity from the reflectors and a low insertion loss. However, the chosen piezoelectric substrate had a temperature coefficient of delay TCD ~69 ppm/°C, which made it very sensitive to temperature variations. Therefore, to compensate for this side effect of the substrate, the authors chose to integrate a temperature sensor on the same chip with the gas sensor. The sensor design was based on a delay line configuration, comprising a bi-directional IDT with uniform finger spacing to provide a symmetrical propagation of the wave in both directions. The IDT had 20 finger pairs, with 2.7 µm finger width (λ/4), and an aperture of 1.08 mm (100 λ). Ten shorted grating reflectors were placed on both tracks and used as follows: two reflectors for each of three sensors (CO_2_, NO_2_ and temperature) and four reflectors for the 4-bits ID tag. For this configuration, the central frequency of the sensors was 440 MHz. The fabrication process started with the deposition of an Al thin film of 150 nm by e-beam evaporation technique. A film of photoresist was then spin-coated, exposed and patterned by photolithography in order to obtain the IDTs and the reflectors. Next, a new layer of poly(methyl methacrylate) (PMMA) of 1 µm thickness was deposited and patterned as passivation layer for the subsequent deposition. ITO with a thickness of 100 nm was then deposited via DC magnetron sputtering. After the ITO patterning, reactive ion etching was used in order to remove the PMMA passivation layer. Next, a thin film of 50 nm of gold was deposited and patterned by the lift-off technique on the area between the first and second reflectors. In this stage, the gold was used as adhesion film between the piezoelectric substrate and the following layer. Then, a transparent film acting as a masking layer was deposited and patterned by photolithography, followed by a new spray-coating of Teflon AF 2400. In the end, the SAW devices were obtained by detaching the transparent film mask.

**Figure 8:**
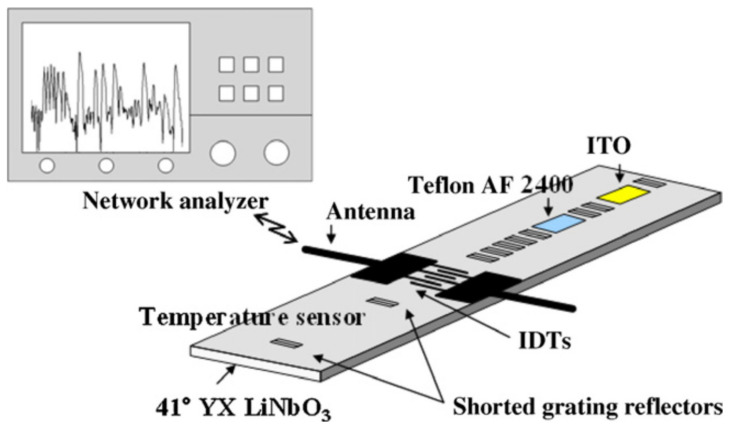
Schematic view of wireless chemical sensor system. Reprinted with permission from Ref. [74]. Copyright 2011 Elsevier.

In addition, two planar antennas were fabricated to enable wireless measurements (Figure 8). One antenna was connected to the fabricated sensor, while the second one was attached to S11 port of the vector network analyzer. A micro heater was also manufactured in order to analyze the effect of temperature variations on sensors’ behavior. ITO was used as a sensing material for NO_2_ detection, benefiting from increased electrical properties than the single oxides own separately. The gas molecules capture the free electrons from the conduction band of the ITO thin film, while a decrease in electrical conductivity and an increase in the propagation velocity of the acoustic wave were observed.

The operation of this sensor was based on changing the propagation speed of the acoustic wave in the deposition zone specific to each analyte. That is why the choice of LiNbO_3_ substrate has the advantage that the propagation of acoustic waves through this material was strongly influenced by the variation of propagation conditions at its surface. The sensor response consisted of a train of radio frequency (RF) pulses whose relative delays to the incident interrogation signal varied depending on the presence of physical and chemical factors in the environment. Afterwards, the delays were converted into phase lags, which were more suggestive when comparing the characteristics of this sensor with the results obtained for other sensor models, measured with VNA. Measurements in the presence of NO_2_ were carried out at 240 °C, at which the maximum sensitivity of the sensor was obtained. The average sensitivity was −51.5°/ppm, as measured over 0.61 ppm to 5 ppm concentration range. Response time was close to 90 s for changing NO_2_ concentration from 0 ppm to 5 ppm, with 900 s recovery time. The sensor section for measuring CO_2_ concentration had a sensitivity of −2.12°/ppm, with the response and recovery times of 40 s and 15 s respectively for 300ppm CO_2_, but the recovery time increased with decreasing CO_2_ concentration. The selectivity of the NO_2_ sensor expressed in phase shift (or signal delay) was 45 times higher than for CO_2_.

Below, Table 3 lists the results obtained in the papers discussed in this chapter, highlighting the most important performances of devices based on the delay line configuration for NO_2_ sensing.

## 6. Summary and Outlook

In this review paper, we have focused on the latest progress in sensing nanomaterials for NO_2_ gas detection using SAW devices. The role of the sensing material is vital in developing SAW gas sensors, aiming to create pores for NO_2_ gas adsorption, or to enlarge the specific surface area with ultra-small nanoparticles that increase the active sites where NO_2_ gas molecules can diffuse. To achieve high performance of the NO_2_ sensors, such as room temperature functionality, low power consumption and flexible devices integration of the sensing nanomaterials, thermal- or photo-activation and utilization of heterojunctions were used as functionalization approaches. From all the nanomaterials investigated, the most sensitive to NO_2_ detection were the colloidal quantum dots, and, moreover, when coupled with MOS nanomaterials, the operational temperature reach room temperature level. Though the metal based CQD require strong acids and organic solvents in their synthesis, and for this reason, the outlook is to develop gas sensors based on graphene or carbon dots, which are the eco-friendly substitute of the CQD.

The SAW device fabrication technology uses standard microelectronic processing techniques, offering high throughput, mass fabrication and reproducibility. The selection of the piezoelectric substrate is a key point in the design of NO_2_ SAW devices with improved performance. As can be seen in Table 2 and Table 3, the piezoelectric substrate most often used for the development of such sensors is quartz (ST cut), due to its excellent properties: crystal quality, low sensitivity to temperature variations and low cost. Other common crystals were LGS, LiTaO_3_ and LiNbO_3_. LGS is mainly used for high-temperature SAW gas applications, and LiTaO_3_ and LiNbO_3_ present pyroelectric proprieties, therefore the electric charge generated by temperature changes limit their processing. LiNbO_3_ presents a high propagation velocity, providing excellent sensitivity to the NO_2_ SAW sensor, and large electromechanical coupling factor K^2^, ensuring low insertion loss. However, it has a relatively high-temperature coefficient of delay TCD, which makes it very sensitive to temperature variations. To overcome this effect, Lim et al. [74] integrated a temperature sensor on the same chip with the NO_2_ SAW device.

Based on the frequency stability of NO_2_ SAW sensors, the most common configuration reported in the literature was the SAW resonator. The phase characteristics’ slope of the oscillators with SAWR is much steeper as compared to delay lines, providing better frequency stability of the SAW gas sensors based on resonator configuration. In addition, this configuration allows the deposition of the NO_2_ sensing material directly over the IDTs, enabling the sensor’s miniaturization.

Improving the characteristics of these sensors requires their characterization as accurately as possible in order to observe every detail of their behavior. Therefore, VNA is considered the ideal instrument for research, because it can measure with very high accuracy and in real-time the amplitude and phase characteristics of any type of device. In addition, broadband VNA operation allows details of sensor characteristics to be observed that cannot be revealed using other types of measurement procedures, such as embedding sensors in oscillators. The circuit characteristics typically used to analyze the performance of two-port sensors consist of variations in the amplitude of the S_21_ transfer coefficient. They allow to highlight the frequency at which a transmission maximum of the S_21_ modulus occurs, as well as changes in this frequency as a function of NO_2_ analyte concentration, as described in [13,31,37,62,86,87,90,92,93,94]. Of the VNA measurements, the best sensitivity was shown in [93], obtaining an RCF deviation of 980 Hz relative to a change in NO_2_ concentration of 1 ppm. However, measuring the sensor response using VNA is not accurate enough if the shape of the |S_21_| characteristic vs frequency has fluctuations in the minimum attenuation region, due to impedance mismatches. The phase characteristic of S_21_ can be used in these scenarios, as described in [62].

Concerns about reducing the cost of measurements and simplifying the processing of experimental data have led to new measurement methods and the use of more affordable laboratory equipment. Therefore, measurement systems using two-port sensors embedded in the positive feedback loop of high-gain amplifiers to obtain oscillators whose operating frequency is dependent on the concentration of the gaseous analyte were reported in several papers [11,33,64]. By combining two sensor-based oscillators, so that only one sensor was covered with a sensing layer, the effect of common-mode measurement errors, due to temperature, humidity and pressure, can be significantly reduced. The operating frequencies of these oscillators can be measured either separately or, after both signals have been fed into a double-balanced mixer, at the IF output of this circuit, using a frequency meter, a low-cost laboratory equipment compared to VNA. Another advantage of this method consists in its very good measurement resolution if the oscillators’ phase noise is small enough at the frequency offset from the carrier corresponding to the desired resolution.

The constructive solution proposed in [74] was used to obtain a sensor for measuring the concentrations of two chemical species (NO_2_ and CO_2_) and the ambient temperature. However, the operating temperature of 240 °C was chosen to maximize the sensitivity of the NO_2_ sensor section. The operating principle of this sensor was based on an SRDL structure with three sensing paths. Since the value of the interrogation signal frequency can be adapted to radio regulations specific to various geographical areas, the device is suitable for wireless applications. The fact that a LiNbO_3_ substrate was used whereby the propagation of acoustic waves was greatly influenced by the environmental conditions at the interface with the environment, has led to a very good sensitivity. The combination of these advantages may contribute to the future commercial success of these category of sensing devices.

## Figures and Tables

**Table 1:** Comparison of coating techniques for the development of gas-SAW sensors.

Coating Technique	Coating Material	Time Efficiency	Cost	Performance at Batch Level	Reproducibility Degree
Drop-casting	Wide range of materials dispersed in liquid media	Individual structure: fast Batch level: slow	Inexpensive	Low	Medium
Spin-coating	Fluids, Polymers	Individual structure: N/A Batch level: very fast	Expensive, requires special equipment	High	High
Material transfer	Carbonaceous, especially graphene	Individual structure: slow Batch level: very slow	Expensive, requires special reagents and clean rooms	Low	Very low

**Table 2:** Selected published work in SAW resonator configuration for NO_2_ detection.

Refs.	Sensing Material	Substrate	Operating Freq. [MHz]	Sensitivity	Response Type	Maximum Linear Response	Limit of Detection (LOD) [ppb]	Response/Recovery Time [s]	Sensor Configuration	Operating Temperature [°C]
[31]	GO-PEDOT-PSS	LGS	135.78	57 Hz/ppm (average for 0 to 100 ppm)	nonlinear (parabolic)	NA	175	35/10	SAWR	RT
[13]	TiO_2_	LGS	135.85	85 Hz/ppm (average for 0 to 100 ppm)	nonlinear (parabolic)	NA	152	310/182	SAWR	RT
	g-C_3_N_4_@TiO_2_	LGS	135.73	197 Hz/ppm (average for 0 to 100 ppm)				143/114		
[86]	ZnO	ST- quartz	99.477	6.56 Hz/ppm (average for 0.4 to 16 ppm)	nonlinear (resonance-like shape)	NA	~400	NA	SAWR	RT
[87]	PZT	ST- quartz	99.193	9.6 Hz/ppm	linear	≥250 ppm ^(1)^	NA	NA	SAWR	RT
[64]	CuPC-MWCNTs	quartz	433.92	7 Hz/ppm (average for 0 to 100 ppm)	partly linear	60 ppm	NA	210/420	SAWR-based dual oscillator	50
	CuPC/ZnO-MWCNTs			48.36 Hz/ppm (avg for 0 to 100 ppm)				500/3000		
[33]	Graphene ink	ST- quartz	262	25 Hz/ppm	linear	≥3 ppm ^(2)^	≤300 ^(2)^	25/35 ^(3)^	SAWR-based dual oscillator	RT
[11]	PANI-WO_3_	ST- quartz	98.47	1.397 Hz/ppm (average for two NO_2_: 77 ppm & 39 ppm)	linear	70 ppm	NA	120/120	SAWR-based dual oscillator ^(4)^	RT
[62]	SWCNTs-CdA	36° YX LiTaO_3_	69.3	0.3°/ppm	linear	8 ppm	160	60/<180	SAWR	RT

Legend: GO-graphene oxide, PEDOT—poly(3,4-ethylenedioxythiophene); PSS—polystyrene sulfonate; ZnO—zinc oxide; CuPC—copper phthalocyanine; MWCNTs—multi-walled carbon nanotubes; PANI-polyaniline; WO3—tungsten oxide; SWCNTs—single-wall carbon nanotubes; CdA—cadmium arachidate; LGS—langasite; LiTaO_3_—lithium tantalate; NA—data not available; RT—room temperature; SAWR: SAW resonator. Numbered notes: ^(1)^ The authors reported measurements over the limited 80–250 ppm NO_2_ concentration range; ^(2)^ These limits are estimated, based on measurements over the limited 0.3–3 ppm NO_2_ concentration range; ^(3)^ Both values were estimated based on the graphs shown in Figure 5 of [30]; ^(4)^ The authors used a dual SAWR (dual SAW resonator), measuring the oscillation frequency with a frequency meter.

**Table 3:** Selected published work in SAW delay line configuration for NO_2_ detection.

Refs.	Sensing Material	Substrate	Operating Freq. [MHz]	Sensitivity	Response Type	Maximum Linear Response	Limit of Detection (LOD) [ppb]	Response/Recovery Time [s]	Sensor Configuration	Operating Temperature [°C]
[37]	PPy-rGO	Y-cut 128° LiNbO_3_	246.99	127.68 Hz/ppm	linear	100 ppm	2370	36.7/58.5	SDL	RT
[94]	Bi_2_S_3_ QDs	ST- quartz	199.86	177 Hz/ppm	linear	10 ppm	17	149/373	SDL	RT
[92]	Untreated PbS QDs	ST- quartz	199.8	20 Hz/ppm	NA			945/813	SPUDT version of SDL	RT
	Treated PbS QDs	ST- quartz		910 Hz/ppm	linear	70 ppm	105	50/60		
[90]	Untreated PbS QDs	ST- quartz	199.9	220 Hz/ppm	linear	10 ppm	32	487/302	SDL	RT
	Treated PbS QDs	ST- quartz		980 Hz/ppm				45/58		
[93]	SnS QDs	ST- quartz	199.78	180 Hz/ppm	linear	10 ppm	52	180/466	SDL	RT
[74]	ITO nanoparticles	41° YX LiNbO_3_	440	51.5°/ppm	linear	75 ppm	NA	90/900	SRDL	240

Legend: GO-graphene oxide, PPy—polypyrrole; QDs—quantum dots; ITO—indium tin oxide; PbS—Lead (II) sulfide; CdS—Cadmium sulfide; SnS—tin sulfide; Bi_2_S_3_—bismuth sulfide; PZT—Lead Zirconium Titanate; WO_3_—tungsten trioxide; g-C_3_N_4_@TiO_2_—graphitic carbon nitride; LiNbO_3_—lithium niobate; NA—data not available; RT—room temperature; SDL: SAW delay line; SRDL SAW reflective delay line.

## Data Availability

Not applicable.
